# Fluorescent Ligand Equilibrium Displacement: A High-Throughput Method for Identification of FMN Riboswitch-Binding Small Molecules

**DOI:** 10.3390/ijms25020735

**Published:** 2024-01-05

**Authors:** Elizabeth D. Tidwell, Ingrid R. Kilde, Suada Leskaj, Markos Koutmos

**Affiliations:** 1 Program in Biophysics, University of Michigan, Ann Arbor, MI 48109, USA; etidwell@umich.edu; 2 Program in Chemical Biology, University of Michigan, Ann Arbor, MI 48109, USA; ikilde@umich.edu; 3 Department of Chemistry, University of Michigan, Ann Arbor, MI 48109, USA; leskajsu@umich.edu

**Keywords:** high-throughput, riboswitch, screening

## Abstract

Antibiotic resistance remains a pressing global concern, with most antibiotics targeting the bacterial ribosome or a limited range of proteins. One class of underexplored antibiotic targets is bacterial riboswitches, structured RNA elements that regulate key biosynthetic pathways by binding a specific ligand. We developed a methodology termed Fluorescent Ligand Equilibrium Displacement (FLED) to rapidly discover small molecules that bind the flavin mononucleotide (FMN) riboswitch. FLED leverages intrinsically fluorescent FMN and the quenching effect on RNA binding to create a label-free, in vitro method to identify compounds that can bind the apo population of riboswitch in a system at equilibrium. The response difference between known riboswitch ligands and controls demonstrates the robustness of the method for high-throughput screening. An existing drug discovery library that was screened using FLED resulted in a final hit rate of 0.67%. The concentration response of each hit was determined and revealed a variety of approximate effective concentration values. Our preliminary screening data support the use of FLED to identify small molecules for medicinal chemistry development as FMN riboswitch-targeted antibiotic compounds. This robust, label-free, and cell-free method offers a strong alternative to other riboswitch screening methods and can be adapted to a variety of laboratory setups.

## 1. Introduction

Worldwide, pathogenic bacterial infections killed 7.7 million people in 2019 [1]. Despite the continued development of antibiotics since the discovery of penicillin in 1928 and the wide availability of antibiotics, bacterial infections cause 13.6% of deaths globally [1]. As a result, bacterial infections continue to be one of the most significant health concerns, especially considering the rise in resistance to current antibacterial or antibiotic drugs [1,2,3,4]. According to a 2019 report by the Centers for Disease Control and Prevention, antibiotic development has stagnated, with only 32 antibiotics under development for bacteria that pose the greatest threats to human health. Out of these, six molecules have been classified as innovative, representing new chemical classes, novel mechanisms of action, or absence of identified cross-resistance [1,2,3]. Identifying new targets for antibiotic development, developing novel methods to identify molecular scaffolds with antimicrobial properties, and proper antimicrobial stewardship worldwide are imperative.

Current antibiotics target bacterial proteins or ribosomes [5,6,7]. In the last 10 years, structured RNA molecules that are not part of the ribosome have joined the discussion as potential antibiotic or antimicrobial targets [5,8,9,10]. The validity of RNA as a novel antibiotic target is supported by research that revealed ribosome-targeting antibiotics that bind specifically to rRNA, not ribosome accessory proteins [6]. This illustrates that RNA-binding small molecules (SMs) can be selective for their targets and can be used with minimal off-target effects. An emerging antibacterial target class is bacterial riboswitches, structured RNA elements that regulate the transcription of specific genes or translation of specific gene products [10,11,12,13]. While riboswitches are present in all domains of life, they are over-represented in bacteria [9,14,15]. Most riboswitches are cis-regulatory, with the riboswitch sequence located 5′ of the gene it regulates; typically, their ligand is intrinsically related to the genes 3′ of the riboswitch [9,12]. Most WHO-priority pathogens, bacteria that pose the greatest threats to human health, contain riboswitches that regulate key biosynthetic pathways [12]. In 2015, ribocil, a compound that inhibited bacterial growth by binding to a riboswitch, was identified using a phenotypic screen. This discovery caused a surge of interest in riboswitches as an antibacterial target [16]. However, we have not seen riboswitch-specific drug candidates progress past early laboratory mouse trials despite some promising results [17,18]. Such work indicates a need to discover new compounds with the potential for development as antibacterial drugs. To discover compound structures, or scaffolds, that can aid in creating new antibiotics through medicinal chemistry refinement, we have established a cell-free, in vitro method to efficiently and rapidly screen SMs that interact with the flavin mononucleotide riboswitch.

Flavin mononucleotide (FMN) plays a crucial role as a cofactor in numerous biosynthetic pathways and serves as a common ligand for a specific riboswitch class [15,19]. FMN riboswitch (FRS) sequences are highly conserved and are present in in 41 out of 49 priority bacterial classes, making them compelling targets for antibiotic development [12,15,20,21]. Additionally, given the already existing foundational FRS biochemical characterization and identification of non-native ligands that can target FRS, we can utilize these previously identified biomolecules as controls to validate our experimental design [16,18,20,22,23].

Assays previously used to identify novel riboswitch ligands include cell-based growth assays, detectable reporter systems, and methods that directly monitor ligand binding or riboswitch cellular function. The most common techniques to identify any antibiotic compound are cell-based assays, specifically those searching for a phenotype rather than a specific interaction, like the bacterial growth inhibition assay used to discover ribocil [16,24,25,26,27,28]. Other cellular methods utilize detectable reporters specifically designed for the riboswitch of interest. These systems typically have a fluorescent or bioluminescent protein sequence downstream from the riboswitch to monitor changes in fluorescence or luminescence as a measure of riboswitch activation or inactivation [29,30,31]. While these systems are robust, they typically require specific bacterial strains and growing conditions, and they often fail to identify compounds that possess activity against the target but are impermeable to the cellular membrane or prone to efflux. These overlooked molecules could potentially be optimized for activity in bacterial cells and, therefore, represent a potential source of additional chemical starting points. Methods involving labeled RNA often utilize a fluorescently tagged RNA scaffold that produces a signal change upon interaction with a ligand [32,33]. This method requires detailed knowledge of the three-dimensional structure of the RNA and extensive controls to ensure that the fluorescent tags do not interfere with the nascent interactions within the RNA or between the RNA and ligand. Even with careful planning, the presence of the label may bias the results.

**Figure 1:**
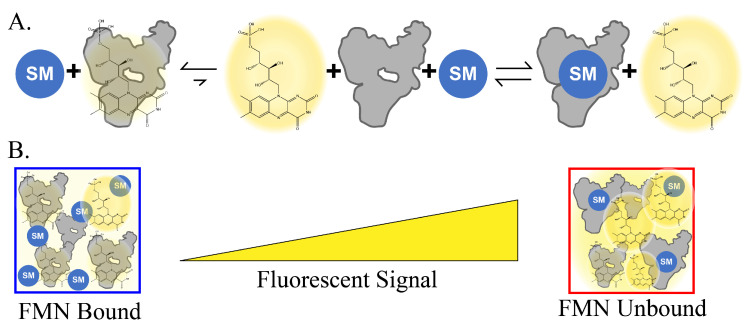
Application of FLED on FRS. Illustration depicting the quenching effect of FMN in the presence of FRS and its utility for for the FLED assay development. (**A**) Equilibrium equation between FMN bound and unbound states in the presence of FRS and another FRS ligand. Blue circles with “SM” represent small molecules ligands, the gray shape represents FRS, and the chemical structure of FMN is highlighted in yellow. (**B**) Pictographic representation of the fluorescent output as FMN unbinds FRS.

**Figure 2:**
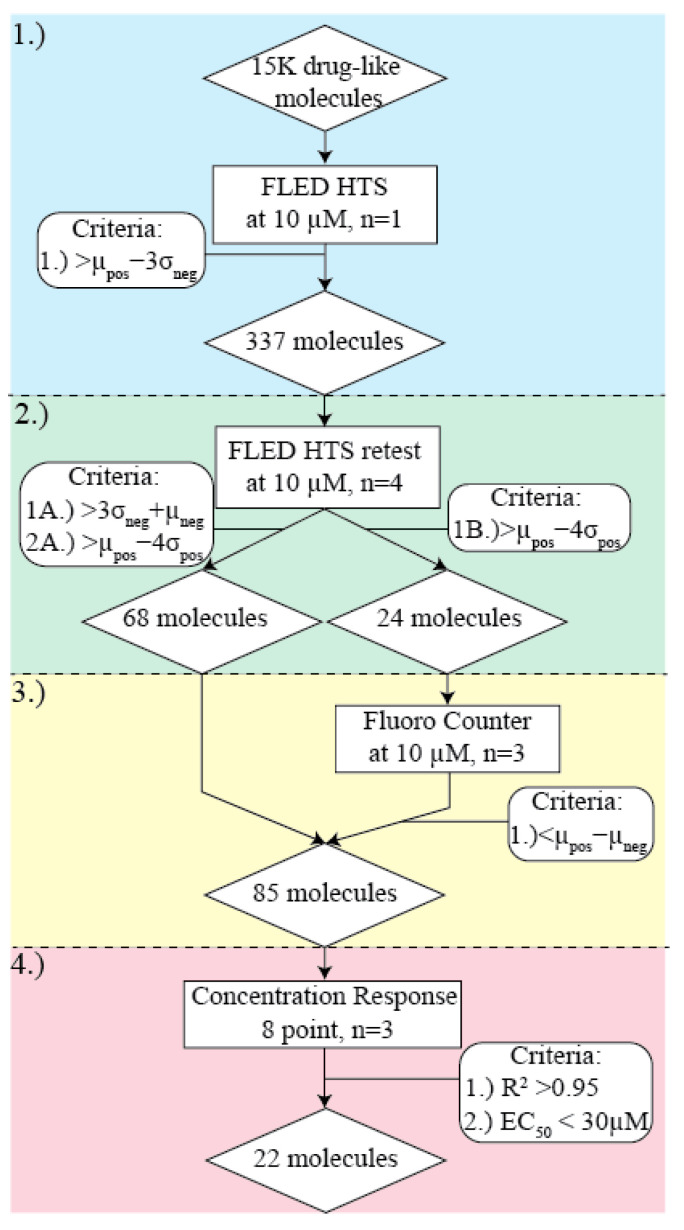
Color-coded workflow for FLED screening of Dart Small Molecule Library. The number of compounds are found in diamonds, methods are written in rectangles, and the criteria for passing into the next phase are listed in rounded boxes. In this figure, μ represents the average fluorescent signal, σ represents the standard deviation of the fluorescent signal, and the sample set is denoted by the subscript (pos = ribocil positive control, neg = DMSO negative control).

Similarly, fluorescently labeled ligands have been used for some riboswitches to monitor the displacement of a native ligand in the presence of other potential binding partners [34,35]. Such a process can be complex since it requires large amounts of the fluorescent ligand and knowledge of the binding mode of the native ligand to design the fluorescent probe molecule. Due to these limitations, we leveraged the inherent fluorescence (Figure 1) of FMN to develop Fluorescent Ligand Equilibrium Displacement (FLED) as a high-throughput, label-free method to identify novel, structurally distinct molecules that bind to the FRS (Figure 2). Hits from FLED screening can be further developed into antibacterial compounds or sensor molecules for fundamental chemistry research.

## 2. Results and Discussion

### 2.1. Considerations for HT Riboswitch Screening (Assay Principle)

In order to rapidly screen for SMs that bind to FRS, it is necessary to consider the scope and context of the screening. The assay was developed to be completely in vitro and cell-free to maximize the number of compounds we could screen, to reduce cost of the assay, and to identify as many hit SMs as possible. As an alternative to using label-based approaches [32,33,36,37], we chose a specific riboswitch with an intrinsically fluorescent native ligand, FMN. Upon interaction with the FRS, forming an FMN–RNA complex, the fluorescence of FMN is modestly quenched. This property of FMN allows for all screening to be based on the increase in FMN fluorescence when it is unbound to the riboswitch (Figure 1).

Although other groups have used this property to monitor the competition of FMN with ligands [16,38], the method has not been optimized as a primary high-throughput screening method. Pure FRS can be easily and efficiently created using traditional in vitro transcription, and both FMN as well as the positive control compound ribocil are commercially available. By using small volumes and relatively inexpensive reagents, this assay is quite cost-effective. Additionally, optimized incubation times and rapid plate reading allow for multiple assays to be performed in parallel. Taken together, this assay is highly scalable for a variety of laboratory environments.

### 2.2. Fluorescent Ligand Equilibrium Displacement Development and Assay Optimization

Through systematic testing of FMN to FRS ratios and RNA concentrations, the optimum background and signal increase upon unbinding was achieved using a 1:2 stoichiometric ratio, FMN to FRS, with a final concentration of 1.5 μ M FRS (Table S1, Figures S1 and S2). The determined ratio and concentration decreased the baseline fluorescence and lowered the standard deviation between samples in the experimental assay. We tested multiple well plates and sample volumes, and the best results with the lowest sample requirements were achieved using 384-well microplates (Corning, Product number 4514). well-plates and a sample volume of 10 μ L. For our positive control, we chose to use the modified compound ribocil-C due to its increased binding affinity compared to ribocil [16]. In all further writing, ribocil refers to ribocil-C. Primary and validation screens were performed using an SM or positive control concentration of 10 μ M.

**Figure 3:**
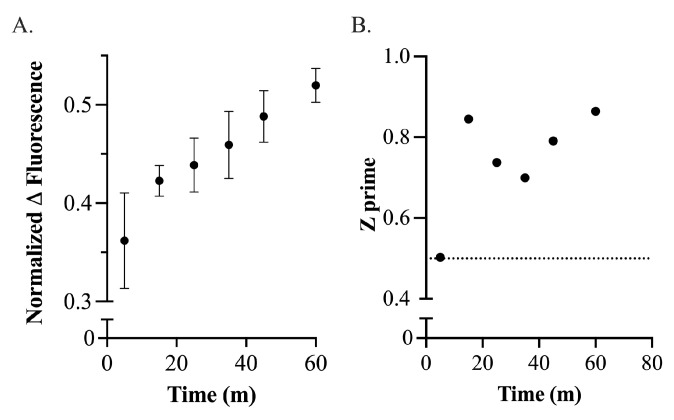
Effect of incubation time on the difference in fluorescent signal and Z’ value. (**A**) When incubating 1.5 μ M FRS with 0.75 μ M FMN and 10 μ M ribocil-C, the change in fluorescence increases with incubation time. (**B**) Calculation of the Z’ score at different incubation times demonstrates that the Z’ value remains above 0.65 after incubating for 15 min. The dashed line represents the Z’ cutoff for an acceptable high-throughput method [39,40,41].

Once the sample concentrations were determined, the difference in fluorescence signal was further optimized by varying the incubation time of the compounds with the FMN–RNA complex (Figure 3). In order to minimize the effect of RNA degradation through magnesium-catalyzed RNA hydrolysis, we chose to limit incubation times to 60 min. We verified that samples incubated for this time range exhibited minimal RNA degradation (Supplementary Figure S2). To determine the minimal incubation time required for acceptable assay performance, we measured the change in fluorescence between positive and negative controls and calculated the assay Z’ at various incubation times under one hour (Figure 3). Z’ represents the statistical difference between positive and negative controls while taking the deviation of each into account and is a measure of assay robustness [39,40,41,42]. Because incubation times greater than 15 min showed a Z’ value well above 0.5, the standard Z’ cutoff for high-throughput assays, we chose an incubation time between 30 and 45 min, with an average incubation time of 37 min, for all subsequent experiments. Incubation time was not increased beyond 60 min due to the time required for the system to reach equilibrium; the longer time period increases the risk of RNA degradation and subsequent false positives.

**Figure 4:**
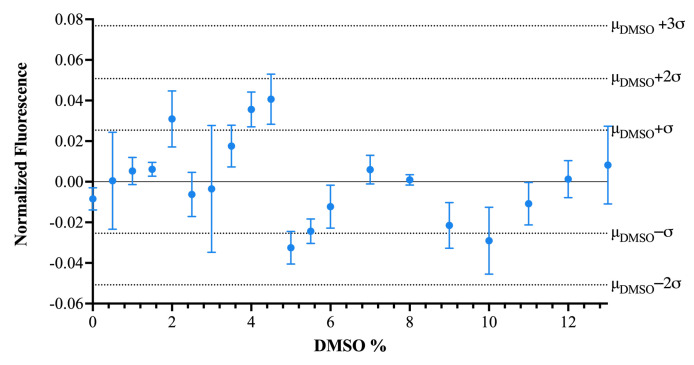
Effect of DMSO percentage on background fluorescent signal.Each dashed line represents the average signal of wells containing 0.5% DMSO plus or minus the specified number of standard deviations across all 0.5% DMSO samples. Blue dots indicate the average signal for the DMSO percentage and the standard deviation is shown with error bars. The sample plate was scanned after 25 min incubation and all wells were prepared using the same RNA preparation.

DMSO is known to have a mild denaturing effect on RNA [43], which could limit the ability of the assay to tolerate high DMSO concentrations, as structural destabilization could produce a false positive due to FMN release. We, therefore, performed a DMSO tolerance test, which demonstrated that the background fluorescence was insensitive to DMSO concentrations up to 13% (Figure 4). These data demonstrate that the DMSO concentrations used (5% for concentration response testing and 0.5% for all other screening) were well tolerated by our assay, and the DMSO concentration could be increased further to accommodate higher SM concentrations or compounds with lower miscibility.

**Figure 5:**
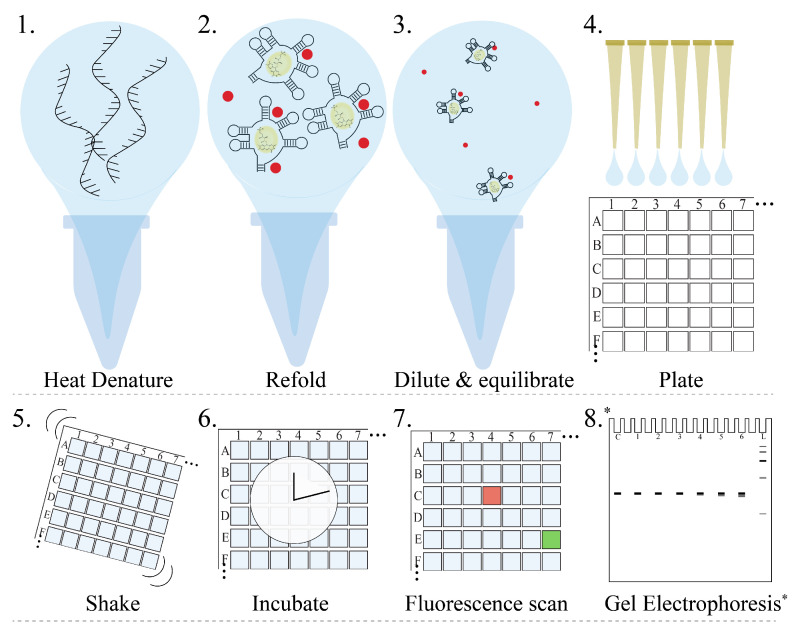
Pictographic representation of FLED experimental procedures. Each step of the process is numbered 1–8. Assessment of RNA quality using gel electrophoresis (step 8) is optional and marked with *. The red spheres in the image represent magnesium ions included for correct folding of the FRS (Supplementary Figure S3).

In the work presented here, the FRS:FMN complex was plated using a manual multiple-channel repeat-dispensing pipette; however, further optimization using more automated systems, such as liquid handlers similar to Biomek FX, could be performed to expedite the experimental setup further. The experimental FLED workflow is illustrated in Figure 5.

### 2.3. Hit Confirmation and Counter Screening

To test our method using an SM library, we screened approximately 15k diverse, drug-like compounds from a collection maintained by the University of Michigan Center for Chemical Genomics. The compounds were screened across multiple days and multiple RNA preparations, with an average Z’ value of 0.805 ± 0.09, a score typically considered excellent for high-throughput screening [39,40,41]. The screening was completed in four phases, each with specific hit thresholds chosen to maintain as many viable hit compounds as possible (Figure 2). Phase 1 experiments are intended to identify any molecules that interact with FRS, but with a higher margin of error. Phase 2 experiments remove experimental false positives and separate compounds that may posses properties that make them incompatible with binding analysis by FLED. Phase 3 identifies and removes compounds that are sufficiently intrinsically fluorescent to be unable to identify any unbinding of FMN from the FRS. Phase 4 assesses if compounds show an SM concentration- or dose-mediated unbinding of FMN.

**Figure 6:**
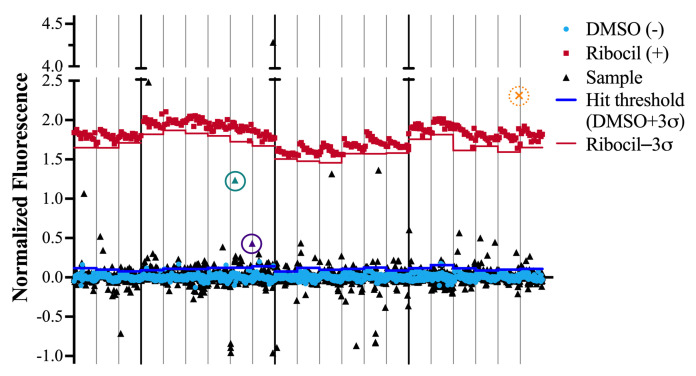
Representative Phase 1 FLED screening data. Each 384 well-plate is denoted with a gray line, and a vertical solid line separates each testing set. Each plate data set was normalized to the average signal from positive control wells, which contain no SM but the same DMSO percentage, 0.5%. Positive control wells (DMSO (−) blue dots) are plotted for each plate, marked by a gray line. Different screening batches are denoted by black vertical lines. Positive control wells, containing ribocil, (Ribocil (+) red squares) are plotted along with a solid red line representing three standard deviations below the average positive control. Each individual compound tested is represented by a single triangle or X symbol. Phase 1 hit criterion is signal DMSO + 3 σ . Select hits present in the following figures are represented with another shape, circled, and are a different color–a purple triangle, a green triangle, and an orange X.

**Figure 7:**
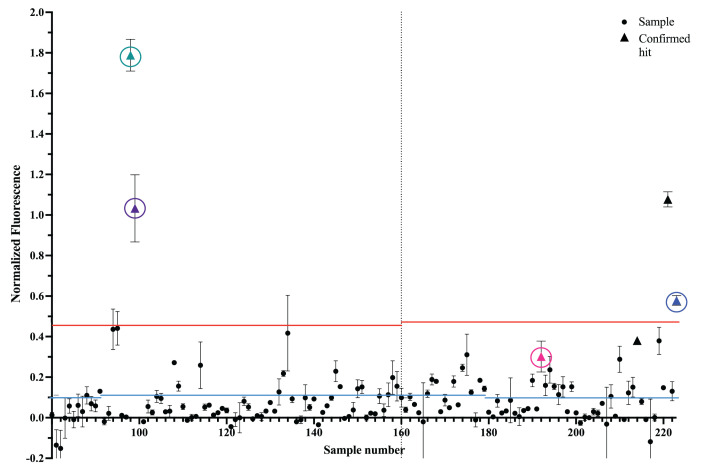
Representative quadruplicate Phase 2 FLED screening. Separate plates are denoted by a vertical dashed line and the average fluorescent signal for each SM, across four replicates, is shown with dots. The standard deviation between the SM is shown with black error bars. Compounds confirmed as hits in subsequent testing are displayed as triangles. The blue dashed line denotes 1A hit threshold ( $3\sigma_{neg}+\mu_{neg}$ ) and the red line represents the 1B intrinsic fluorescence threshold ( $\mu_{pos}-4\sigma_{pos}$ ). Compounds above the red line were tested in Phase 3, while compounds between the blue and red lines were advanced directly to Phase 4. The black points are the average signal of each molecule, with the standard deviation between each well as the error bars. Select hits present in the preceding or following figures are represented with another shape, circled, and are represented with a different color–a purple triangle, a teal triangle, a pink triangle, and a blue triangle.

Specifically, Phase 1 consisted of single-replicate screening of each compound using FLED. Any compounds meeting the hit criterion (a fluorescence signal at least three standard deviations above that of the average negative control) were advanced to Phase 2 of screening (Figure 6). In Phase 2, each compound was retested at the same concentration in triplicate or quadruplicate in order to account for the inherent variability in high-throughput screening (Figure 7). The hit criterion and the Z’ calculations were chosen based on the negative control rather than the mean of all samples in order to avoid the contributions of false positives due to inherent properties of the compounds tested (such as intrinsic fluorescence or aggregation) artificially increasing the hit threshold. During this phase, compounds were sorted into two hit categories based on satisfying criteria 1A or 1B (Figure 2). Without further testing, molecules with fluorescent signals between criteria 1A and 2A (Figure 2) were advanced to Phase 4. However, compounds with a signal above criterion 1B (Figure 2) were tested in Phase 3. The high fluorescent signal in these cases could be due to intrinsic fluorescence, not equilibrium competition with FMN. Unlike Phases 1 and 2, Phase 3 tested compounds using an altered version of FLED. Each molecule was plated for a final concentration at 10 μ M as before, but the buffer without FRS or FMN was added instead of the FMN:FRS complex to each compound well.

**Figure 8:**
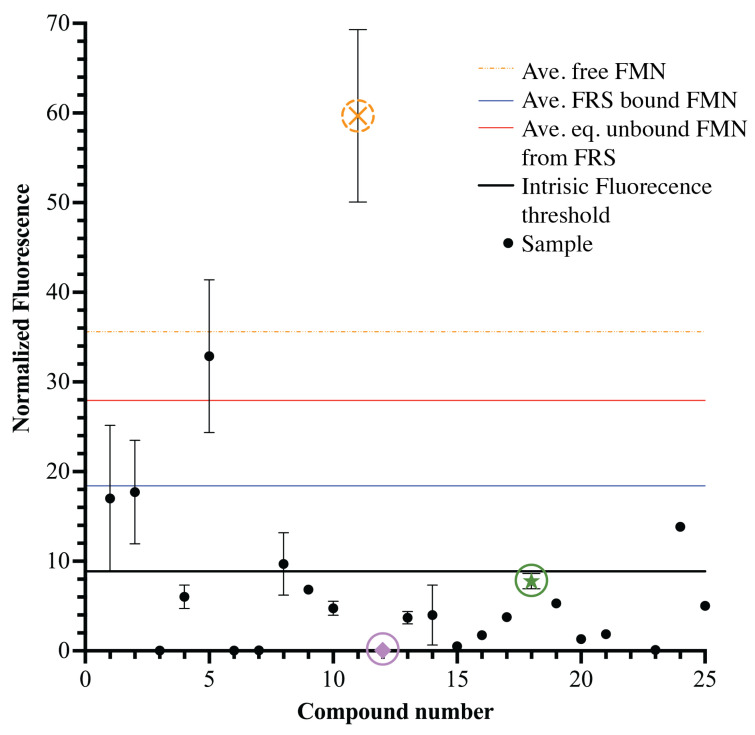
Fluorescence counter screen, Phase 3 FLED screening. Assay to assess the intrinsic fluorescence of SMs flagged during Phase 2 FLED screening. The black points with whisker error bars represent the average fluorescent signal of an individual compound with the standard deviation between each sample well. A compound from Figure 6 is highlighted with an X symbol and a dashed orange circle as one removed from further analysis due to intrinsic fluorescence. The fluorescence threshold (solid black line) is defined as $\mu_{pos}-\mu_{neg}$ . Each solid line represents the fluorescent signal, averaged across 8 replicates, of FMN bound to FRS, FLED negative control; equilibrium population of unbound FMN from FRS, positive control; and free FMN, no RNA present. Select hits present in the preceding or following figures are represented with another shape, circled, and are represented with a different color–a lavender diamond, a green star, and an orange X.

Compounds with a signal greater than the difference between positive and negative controls were flagged as intrinsically fluorescent and removed from screening, while those below the threshold advanced to Phase 4 (Figure 8). Using this counter screen, we removed molecules so intrinsically fluorescent that binding was undetectable using FLED while retaining intrinsically fluorescent molecules that could bind to the FRS. Compounds that fit the criteria for Phases 1–3 were considered confirmed hits and moved into Phase 4—concentration response testing.

### 2.4. Confirmed Hit Concentration–Response Testing

**Figure 9:**
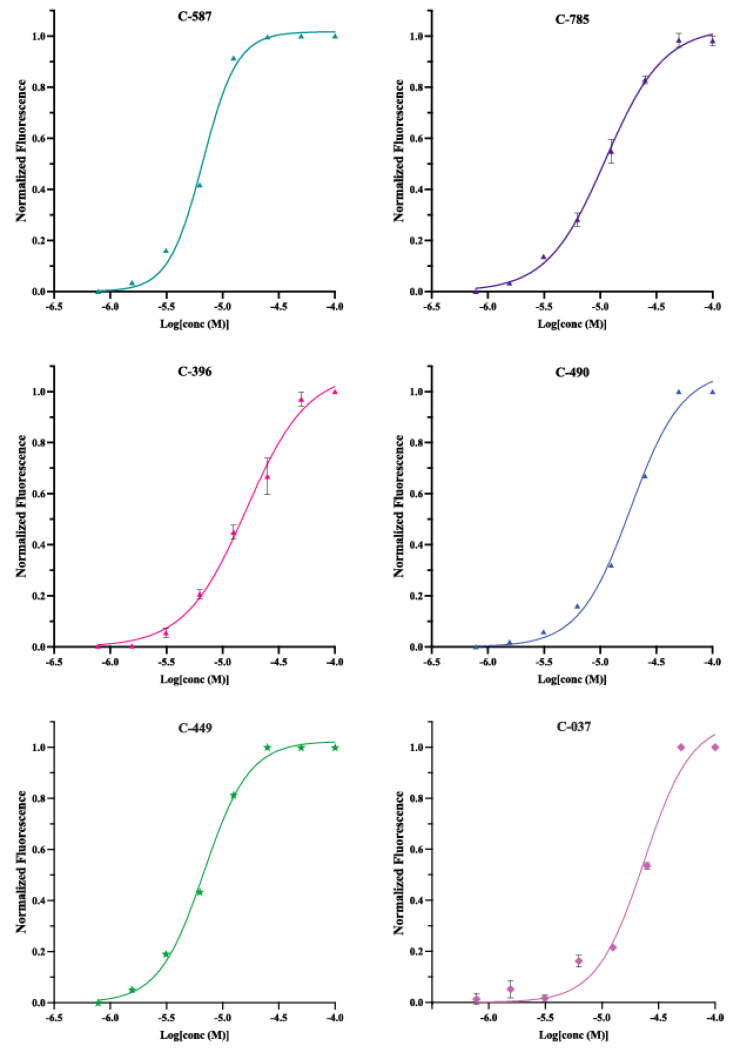
Concentration–response curves of highlighted Phase 4 hits. All data sets were normalized to a maximum signal of 1 and a baseline value of 0. Each square point represents the average signal across 3 replicates; standard deviation is shown with the cross bars. Non-linear regression curves, Hill slopes, goodness of fit, and EC_50_ were calculated using Prism 10, sigmodial dose response (variable slope) analysis program (Materials and Methods). Select hits present in the preceding figures are represented with a unique color and shape combination–purple triangle, a teal triangle, a blue triangle, a pink triangle, a lavender diamond, and a green star.

In Phase 4, compounds were plated in a two-fold decreasing concentration series, from 100 μ M to 0.78 μ M (Supplementary Table S2). Each compound was tested in quadruplicate and analyzed using the default parameters in MScreen [42]. Similar methods have been used previously to describe the binding of molecules to the FMN riboswitch [38]. Compounds with non-negative Hill slopes and R squared values above 0.8 were further analyzed using Prism 10 to identify the approximate EC_50_, or half-maximal effective concentration required to reach SM specific maximum FMN release, along with improved Hill slope calculations and goodness of fit, R^2^ (Section 3). We restricted our fully validated hits to compounds with an R^2^ value greater than 0.95 and an EC_50_ value less than 30 μ M (Figure 9, Table 1, Supplementary Figure S5). Using these parameters, we identified 22 compounds with concentration–response using the four-phase FLED workflow (Figure 2). Each of these validated hits underwent further analysis using low-throughput methods such as isothermal calorimetry and transcription termination assays for efficacy beyond the FLED system. Across the 22 hits, 4 of the EC_50_ values were between 1 μ M and 10 μ M, 10 were between 10 μ M and 20 μ M, and 7 were between 20 μ M and 30 μ M.

## 3. Materials and Methods

### 3.1. RNA In Vitro Synthesis and Purification

In vitro transcribed FRS was prepared using T7 RNA polymerase [44,45]. The T7 polymerase was purified in-house using established protocols [44,45,46]. DNA template oligos were purchased from Integrative DNA Technologies (IDT) as single-stranded DNA (Supplementary Table S1). The template consisted of the antisense sequence of the RNA desired, with the first two nucleotides replaced with 2’ O-methylated nucleotides to improve RNA 3’ end homogeneity [47,48].

Before transcription, the DNA template was mixed with DNA oligo coding for the T7 promoter sequence and allowed to sit at room temperature for at least two minutes. DNA template and T7 promoter oligo final concentration was 0.1 μ M each. Transcription reactions were performed in 40 mM tris(hydroxymethyl)aminomethane (Tris) pH 8, 0.01% Triton-X, 30 mM magnesium chloride (MgCl_2_), 7.11 mM ATP, 7.71 mM CTP, 10.07 mM GTP, 7.11 mM UTP, 10 mM DTT, 2 mM spermidine, 0.5 U/mL inorganic pyrophosphatase (purchased from ThermoFisher Scientific), 3% dimethyl sulfoxide (DMSO), and 0.7 μM T7 RNA polymerase. Reactions were allowed to proceed for 3.75 to 4 hours at 37 °C while being shaken at 300 rpm and then quenched by adding EDTA, pH 8.0, to a final concentration of 60 mM. Samples were flash-frozen and then stored at −80 °C overnight before purification by size exclusion chromatography.

Transcription reactions were thawed, and then the buffer was exchanged using Amicon 30 kDa molecular weight cutoff filters into the SEC buffer (5 M Urea, 90 mM Tris base pH 7, 90 mM boric acid, 2 mM EDTA). Samples were concentrated to an approximately 0.5 mL volume, which is appropriate for injection onto a Fast Pressure Liquid Chromatography (FPLC) system. Before direct injection, samples were filtered through 0.22 µm SpinX filters, then injected onto an equilibrated Superdex 200 Increase 10/300 column (GE Healthcare, Chicago, IL, USA, now Cytiva). Samples were isocratically eluted at a flow rate of 0.3–0.4 mL/min at 4 °C and collected into 0.4 mL fractions.

Before pooling and buffer exchange into storage buffer, samples within the major RNA product peak, around 13 mL, were analyzed on a 12% denaturing polyacrylamide gel to ensure the FRS RNA was of the correct molecular weight and free of nucleotide contamination. Samples of appropriate purity (Supplementary Figure S4) were pooled. Then, the buffer was exchanged into 50 mM Tris pH 6.5, 50 mM boric acid, and 150 mM potassium chloride (KCl) using a fresh equilibrated Amicon 30 kDa spin concentrator and stored at −80 °C.

### 3.2. Refolding and FMN Complex Formation

All procedures involving FMN were performed in low-light conditions to prevent photobleaching. All FMN solutions were prepared fresh from powder (Sigma Aldrich, St. Louis, MO, USA).

Frozen FRS RNA was thawed gently on ice, was heat denatured at 90 °C for two minutes, and was then immediately diluted into a room temperature solution of 50 mM Tris pH 6.5, 50 mM boric acid, 150 mM KCl, 3.75 μ M FMN, and 10 mM MgCl_2_ in a foil-wrapped tube, to an FRS concentration of 7.5 µM. The FRS was allowed to fold at 37 °C for 20 min, then further diluted in 50 mM Tris pH 6.5, 50 mM boric acid, and 150 mM KCl to a final concentration of 1.5 μ M FRS, 0.75 μ M FMN, and 2 mM MgCl_2_. This solution was incubated at 23 °C (room temperature) for 20 min before use in assays.

### 3.3. Fluorescent Ligand Equilibrium Displacement (FLED)

All procedures involving FMN were performed in low-light conditions to prevent photobleaching. All FMN solutions were prepared fresh from powder (Sigma Aldrich).

During FRS folding, compounds dissolved in DMSO were spotted on Corning 4514 low-volume black plates using an Echo 655 Acoustic Liquid Handler. Primary screening was performed with n = 1 using stock compounds of 2 mM for a final concentration of 10 μ M and a DMSO concentration of 0.5%. Folded FRS solution (10 μ L) was added by hand using a 12-channel repeat-dispensing pipette. Plates were then shaken at 300 rpm on a Thermo Multidrop Combi for 2–3 s, spun down at 201 xg for one minute using a swing bucket centrifuge, then incubated at room temperature for, on average, 37 min (between 30 and 43 min) for an individual plate. Plates were scanned using the BMG PHERAstar plate reader. The gain and volume on each plate were adjusted to a positive control well and scanned at 485 nm, excitation 520 nm, 30 flashes per well. Compounds with fluorescence increase above the hit threshold $\left(\mu_{DMSO}+3\left(\sigma_{DMSO}\right)\right)$ were counted as hits and subjected to secondary testing. Secondary testing was completed in the same manner as above but with compounds plated in triplicate. Compounds that showed an average fluorescent signal increase above the hit threshold $\left(\mu_{DMSO}+3\left(\sigma_{DMSO}\right)\right)$ and below the intrinsic fluorescent threshold $\left(\mu_{ribocil}-4\left(\sigma_{ribocil}\right)\right)$ were then used for dose–response testing.

Compounds with average fluorescent signal above the intrinsic fluorescent threshold $\left(\mu_{ribocil}-4\left(\sigma_{ribocil}\right)\right)$ were subjected to counter-screening to determine if intrinsic fluorescence was high enough to invalidate the previous testing. Compounds were spotted onto plates, 50 nL each, as before, but in quadruplicate. Instead of adding 10 μ L of FRS solution, buffer was added to each compound well. Any compounds with average signal above the fluorescent threshold ( $\mu_{ribocil}-\mu_{DMSO}$ ) were removed from further testing. For this assay, compound fluorescence was normalized to the background fluorescence of the sample buffer containing 0.5% DMSO.

Dose–response testing was completed in the same manner as above, but the selected compounds and DMSO were plated to concentrations ranging from 0.781 μ M to 100 µM on an eight-point curve (Supplementary Table S2). Each compound was tested at each measured concentration in triplicate. SMs with initial Hill slopes above 0, and R^2^ values above 0.8, as reported by the MScreen software [42], were further analyzed using Prism 10 Software. Before analysis, the concentrations were converted to log concentration and all data sets normalized so that the maximum replicate signal was equal to 1 and lowest replicate signal was equal to 0. Each concentration was averaged and the standard deviation was calculated. The resulting information for each SM was analyzed using the nonlinear fit, sigmodal-dose response (variable slope) fitting analysis. The only parameter changed was constraining the baseline value to 0.

Representative raw and normalized data from each phase of screening is provided for two different compounds in Supplementary Table S3.

## 4. Conclusions

After screening approximately 15k compounds, the hit rate dropped from 2.6% in Phase 1 to 0.15% after Phase 4, using stringent final screening criteria. FLED identified 22 structurally distinct small molecules with a low micromolar activity, providing evidence that this system can isolate compounds that span a large chemical space, which may increase the probability of identifying compounds that are less prone to antibiotic resistance. The methodology described above has proven robust enough to identify compounds that are able to bind to the FRS despite the presence of the native ligand, FMN. Each step of FLED can be completed in under an hour, and the process from refolding to the last plate scanned can be completed for 8 plates in approximately 90 minutes using a lower-throughput sample application method. The fundamental principle of the method is grounded in the inherent properties of the interactions between FMN and the riboswitch and, therefore, could be used with other FRS sequences, beyond the *F. nucleatum* construct used here, with minimal optimization. The setup described above can be adjusted to higher (e.g., 1536-well) or lower (e.g., 96-well) density plate formats, or to accommodate fully automated screening systems, further increasing the quantity of compounds screened per day. Moreover, the range of acceptable incubation times allows for this technique to be used with or without rapid scanning technology such as plate readers with attached stackers and accommodates delays due to technological issues. The major parameters that may need to be adjusted are the volume of FMN:FRS complex (if using assay plates that are not optimized for low volume) and incubation time of the plates after adding complex. Also, this method could be used to screen libraries of molecular fragments and reveal fragments capable of interacting in the riboswitch-binding pocket; however, it would be unable to identify hits with very low affinity or ones that interact with cryptic binding sites.

The simplicity of this method, requiring at a minimum a fluorescent plate reader, makes it tenable for both larger highly resourced laboratories and smaller research groups. This scalability is particularly useful since the majority of antibiotic research carried out today is at smaller start-up biotech companies and academic research institutions [7,49]. FLED, also, could be used by labs without any automation to test natural products or newly synthesized compounds for activity on the FRS. Another advantage of this method is that mutations arise in the sequence of FRS—the new mutant riboswitches can replace the original RNA used for screening. As long as the riboswitch maintains a high-affinity binding interaction with FMN, it can be used for FLED screening. Each scaffold validated in the method can be successively improved using medicinal chemistry parameters in a systematic manner. While identification of FRS-binding small molecules could lead to the identification of novel antibiotics, the number and variety of compounds could easily be used in combination therapies. These applications spread the evolutionary pressure to develop drug resistance across multiple separate molecules. For example, it has been shown that administering multiple drugs can re-sensitize bacteria to drugs and reduce the development of resistance overall [50,51]. The principle of FMN unquenching can even be applied more broadly, in systems outside of FRS, to target FMN-binding proteins in bacteria or humans and may identify compounds with therapeutic effects in diseases other than bacterial infections. FLED is a robust method with great potential for use across many research institutions and applications in multiple biologically relevant systems, beginning with bacterial riboswitches and new antimicrobial discovery.

## Figures and Tables

**Table 1:** Dose response activity of highlighted compounds. Curves and plots are provided in Figure 9.

Compound ID	EC_50_ ( μ M)	Hill Slope	Goodness of Fit (R^2^)
587	6.67	2.84	0.99
785	11.1	1.67	0.99
396	16.3	1.59	0.99
490	18.6	1.89	0.99
449	6.82	2.15	0.99
037	23.6	2.07	0.98

## Data Availability

Representative data is present in the supplementary materials. More data sets presented in this study are available upon request from the corresponding author.

## Supplementary Materials

The following supporting information can be downloaded at: www.mdpi.com/xxx/s1.

## Abbreviations

The following abbreviations are used in this manuscript:

RNA	ribonucleic acid
FMN	flavin mononucleotide
FRS	FMN riboswitch
SM	small molecule
FLED	Fluorescent Ligand Equilibrium Unquench assay
FPLC	Fast Pressure Liquid Chromotography
DMSO	dimethyl sulfoxide

## References

[1] Ikuta K.S., Swetschinski L.R., Robles Aguilar G., Sharara F., Mestrovic T., Gray A.P., Davis Weaver N., Wool E.E., Han C., Gershberg Hayoon A. (2022). Global mortality associated with 33 bacterial pathogens in 2019: A systematic analysis for the Global Burden of Disease Study 2019. Lancet.

[2] WHO (2022). 2021 Antibacterial Agents in Clinical and Preclinical Development: An Overview and Analysis.

[3] Laxminarayan R., Duse A., Wattal C., Zaidi A.K., Wertheim H.F., Sumpradit N., Vlieghe E., Hara G.L., Gould I.M., Goossens H. (2013). Antibiotic resistance—the need for global solutions. Lancet Infect. Dis..

[4] Centers for Disease Control and Prevention (2022). *Outpatient Antibiotic Prescriptions—United States, 2021*; Technical Report. https://www.cdc.gov/antibiotic-use/data/report-2021.html.

[5] Silver L.L. (2016). Appropriate targets for antibacterial drugs. Cold Spring Harb. Perspect. Med..

[6] Lin J., Zhou D., Steitz T.A., Polikanov Y.S., Gagnon M.G. (2018). Ribosome-Targeting Antibiotics: Modes of Action, Mechanisms of Resistance, and Implications for Drug Design HHS Public Access. Annu. Rev. Biochem..

[7] Chokshi A., Sifri Z., Cennimo D., Horng H. (2019). Global Contributors to Antibiotic Resistance. J. Glob. Infect. Dis..

[8] Ecker D.J., Griffey R.H. (1999). RNA as a small-molecule drug target: Doubling the value of genomics. Drug Discov. Today.

[9] Papenfort K., Vogel J. (2010). Regulatory RNA in Bacterial Pathogens. Cell Host Microbe.

[10] Yu A.M., Choi Y.H., Tu M.J. (2020). RNA Drugs and RNA Targets for Small Molecules: Principles, Progress, and Challenges. Pharmacol. Rev..

[11] Deigan K.E., Ferré-D’Amaré A.R. (2011). Riboswitches: Discovery of drugs that target bacterial gene-regulatory RNAs. Accounts Chem. Res..

[12] Pavlova N., Penchovsky R. (2019). Genome-wide bioinformatics analysis of FMN, SAM-I, glmS, TPP, lysine, purine, cobalamin, and SAH riboswitches for their applications as allosteric antibacterial drug targets in human pathogenic bacteria. Expert Opin. Ther. Targets.

[13] Panchal V., Brenk R. (2021). Riboswitches as Drug Targets for Antibiotics. Antibiotics.

[14] Gelfand M., Mironov A., Jomantas J., Kozlov Y., Perumov D. (1999). A conserved RNA structure element involved in the regulation of bacterial riboflavin synthesis genes. Trends Genet..

[15] Mironov A.S., Gusarov I., Rafikov R., Lopez L.E., Shatalin K., Kreneva R.A., Perumov D.A., Nudler E. (2002). Sensing Small Molecules by Nascent RNA: A Mechanism to Control Transcription in Bacteria. Cell.

[16] Howe J.A., Wang H., Fischmann T.O., Balibar C.J., Xiao L., Galgoci A.M., Malinverni J.C., Mayhood T., Villafania A., Nahvi A. (2015). Selective small-molecule inhibition of an RNA structural element. Nature.

[17] Blount K.F., Megyola C., Plummer M., Osterman D., O’connell T., Aristoff P., Quinn C., Chrusciel R.A., Poel T.J., Schostarez H.J. (2015). Novel Riboswitch-Binding Flavin Analog That Protects Mice against Clostridium difficile Infection without Inhibiting Cecal Flora. Antimicrob. Agents Chemother..

[18] Vicens Q., Mondragón E., Reyes F.E., Coish P., Aristoff P., Berman J., Kaur H., Kells K.W., Wickens P., Wilson J. (2018). Structure-Activity Relationship of Flavin Analogues That Target the Flavin Mononucleotide Riboswitch. ACS Chem. Biol..

[19] Vitreschak A.G., Rodionov D.A., Mironov A.A., Gelfand M.S. (2002). Regulation of riboflavin biosynthesis and transport genes in bacteria by transcriptional and translational attenuation. Nucleic Acids Res..

[20] Serganov A., Huang L., Patel D.J. (2009). Coenzyme recognition and gene regulation by a flavin mononucleotide riboswitch. Nature.

[21] Tucker B.J., Breaker R.R. (2005). Riboswitches as versatile gene control elements. Curr. Opin. Struct. Biol..

[22] Vicens Q., Mondragón E., Batey R.T. (2011). Molecular sensing by the aptamer domain of the FMN riboswitch: A general model for ligand binding by conformational selection. Nucleic Acids Res..

[23] Rizvi N.F., Howe J.A., Nahvi A., Klein D.J., Fischmann T.O., Kim H.Y., McCoy M.A., Walker S.S., Hruza A., Richards M.P. (2018). Discovery of Selective RNA-Binding Small Molecules by Affinity-Selection Mass Spectrometry. ACS Chem. Biol..

[24] Fleming A. (1929). On the antibacterial action of cultures of a penicillium, with special reference to their use in the isolation of B. influenzae. Br. J. Exp. Pathol..

[25] Dubos R.J., Hotchkiss R.D. (1941). The production of bactericidal substances by aerobic sporulating bacilli. J. Exp. Med..

[26] Duggar B.M. (1948). Aureomycin: A product of the continuing search for new antibiotics. Ann. N. Y. Acad. Sci..

[27] Finlay A.C., Hobby G.L., P’an S.Y., Regna P.P., Routien J.B., Seeley D.B., Shull G.M., Sobin B.A., Solomons I.A., Vinson J.W. (1950). Terramycin, a New Antibiotic. Science.

[28] Waksman S.A., Schatz A., Reynolds D.M. (2010). Production of antibiotic substances by actinomycetes. Ann. N. Y. Acad. Sci..

[29] Kirchner M., Schorpp K., Hadian K., Schneider S. (2017). An in vivo high-throughput screening for riboswitch ligands using a reverse reporter gene system. Sci. Rep..

[30] Penchovsky R., Pavlova N., Kaloudas D. (2021). RSwitch: A Novel Bioinformatics Database on Riboswitches as Antibacterial Drug Targets. IEEE/ACM Trans. Comput. Biol. Bioinform..

[31] Kennedy K.J., Widner F.J., Sokolovskaya O.M., Innocent L.V., Procknow R.R., Mok K.C., Taga M.E. (2022). Cobalamin Riboswitches Are Broadly Sensitive to Corrinoid Cofactors to Enable an Efficient Gene Regulatory Strategy. mBio.

[32] Chinnappan R., Dubé A., Lemay J.F., Lafontaine D.A. (2013). Fluorescence monitoring of riboswitch transcription regulation using a dual molecular beacon assay. Nucleic Acids Res..

[33] Baird N.J., Inglese J., Ferré-D’Amaré A.R. (2015). Rapid RNA-ligand interaction analysis through high-information content conformational and stability landscapes. Nat. Commun..

[34] Su Y., Hickey S.F., Keyser S.G., Hammond M.C. (2016). In Vitro and in Vivo Enzyme Activity Screening via RNA-Based Fluorescent Biosensors for S-Adenosyl- l -homocysteine (SAH). J. Am. Chem. Soc..

[35] Cai Y., Xia M., Dong H., Qian Y., Zhang T., Zhu B., Wu J., Zhang D. (2018). Engineering a vitamin B 12 high-throughput screening system by riboswitch sensor in Sinorhizobium meliloti. BMC Biotechnol..

[36] Hickey S.F., Hammond M.C. (2014). Structure-guided design of fluorescent S-adenosylmethionine analogs for a high-throughput screen to target SAM-I riboswitch RNAs. Chem. Biol..

[37] Crielaard S., Maassen R., Vosman T., Rempkens I., Velema W.A. (2022). Affinity-Based Profiling of the Flavin Mononucleotide Riboswitch. J. Am. Chem. Soc..

[38] Chen Q., Li Y., Lin C., Chen L., Luo H., Xia S., Liu C., Cheng X., Liu C., Li J. (2022). Expanding the DNA-encoded library toolbox: Identifying small molecules targeting RNA. Nucleic Acids Res..

[39] Zhang J.H., Chung T.D., Oldenburg K.R. (1999). A Simple Statistical Parameter for Use in Evaluation and Validation of High Throughput Screening Assays. J. Biomol. Screen..

[40] Sui Y., Wu Z. (2007). Alternative Statistical Parameter for High-Throughput Screening Assay Quality Assessment. Slas-Discovery.

[41] Birmingham A., Selfors L.M., Forster T., Wrobel D., Kennedy C.J., Shanks E., Santoyo-Lopez J., Dunican D.J., Long A., Kelleher D. (2009). Statistical methods for analysis of high-throughput RNA interference screens. Nat. Methods.

[42] Jacob R.T., Larsen M.J., Larsen S.D., Kirchhoff P.D., Sherman D.H., Neubig R.R. (2012). MScreen: An integrated compound management and high-throughput screening data storage and analysis system. J. Biomol. Screen..

[43] Lee J., Vogt C.E., McBrairty M., Al-Hashimi H.M. (2013). Influence of Dimethylsulfoxide on RNA Structure and Ligand Binding. Anal. Chem..

[44] Milligan J.F., Groebe D.R., Witherell G.W., Uhlenbeck O.C. (1987). Nucleic Acids Research Oligoribonucleotide synthesis using T7 RNA polymerase and synthetic DNA templates. Nucleic Acids Res..

[45] Milligan J.F., Uhlenbeck O.C. (1989). Synthesis of small RNAs using T7 RNA polymerase. Methods Enzymol..

[46] Brunelle J.L., Green R. (2013). In Vitro Transcription from Plasmid or PCR-amplified DNA. Methods Enzymol..

[47] Kao C., Zheng M., Rüdisser S. (1999). A simple and efficient method to reduce nontemplated nucleotide addition at the 3 terminus of RNAs transcribed by T7 RNA polymerase. RNA.

[48] Kao C., Rüdisser S., Zheng M. (2001). A simple and efficient method to transcribe RNAs with reduced 3’ heterogeneity. Methods.

[49] Renwick M., Mossialos E. (2018). What are the economic barriers of antibiotic R&D and how can we overcome them?. Expert Opin. Drug Discov..

[50] Basavegowda N., Baek K.H. (2022). Combination Strategies of Different Antimicrobials: An Efficient and Alternative Tool for Pathogen Inactivation. Biomedicines.

[51] Singh N., Yeh P.J. (2017). Suppressive drug combinations and their potential to combat antibiotic resistance. J. Antibiot..

