# Supplementary material for: Fluorescent Ligand Equilibrium Displacement: A High-Throughput Method for Identification of FMN Riboswitch-Binding Small Molecules
Source: Int J Mol Sci. 2024 Jan 5;25(2):0. doi: 10.3390/ijms25020735 (PMC11154562; doi:10.3390/ijms25020735)
Supplement: Supplementary file 1 [file ijms-25-00735-s001.zip › ijms-2708042-supplementary.pdf]

Supplementary materials

Fluorescent Ligand Equilibrium Displacement: a high-throughput method for identification of FMN riboswitch binding small molecules

Elizabeth D Tidwell, Ingrid R Kilde, Suada Leskaj, and Markos Koutmos

Information on the optimization of FLED experimental conditions.

Initial work was done using a variety of FMN concentrations and RNA concentrations. This initial optimization used 96 well plates but then switched to 384 low volume, which was used for FLED screening. First, we tested the intrinsic fluorescence of FMN (Figure S1 A), the background fluorescence of FMN when in complex with FRS (Figure S1 B), and the fluorescence increase upon denaturation by adding 50% DMSO, values plotted had the initial background fluorescence from the previous scan without DMSO(Figure S1 C). Each of these scans was gain-adjusted to the highest free FMN well. We retested this process and found again that the 2:1 ratios appeared more consistent and that the increase was less when the RNA concentration was greater than 2 uM (Figure S1 D). So, we chose to use the 2:1 RNA to FMM ratio at an RNA concentration of 1.5 uM.

We further tested this concertation in the presence of ribocil, using 10 uM as the final concertation since it is commonly used as an initial screening concertation. We extensively tested the effects of time and DMSO on the complex described in the main text. We also validated the ability of ribocil to act as a negative control. Across a control plate with many replicates of negative control, DMSO without small molecules, and positive control, DMSO with ribocil, and calculated a Z' value of 0.82 (Figure S2). All optimization and testing was performed using the sequences listed in Table S1.

Table S1. Relevant sequences used for in vitro transcription of FMN riboswitch (FRS). A lowercase m preceding a nucleotide indicates a 2' O-methylation modification on the ribose of the nucleotide.

Oligo name	Oligo sequence 5' to 3'
T7 promoter clamp (DNA)	TAA TAC GAC TCA CTA TAG G
FRS anti-sense (DNA)	mGmAA TCT TCT CTC ATC CAG ACT CTA CTG TCG GTT TTG GAA TTT CAC CAA ATC AAA GCA AAT ACT TTC GTG GAC TAT ACC ACC GGT CGG GAA TTT CAC CCT GCC CCG AAG ATC CTA TAG TGA GTC GTA TTA
FRS sense (RNA)	G GGA UCU AUC UUC GGG GCA GGG UGA AAU UCC CGA CCG GUG GUA UAG UCC ACG AAA GUA UUU GCU UUG AUU UGG UGA AAU UCC AAA ACC GAC AGU AGA GUC UGG AUG AGA GAA GAU UC

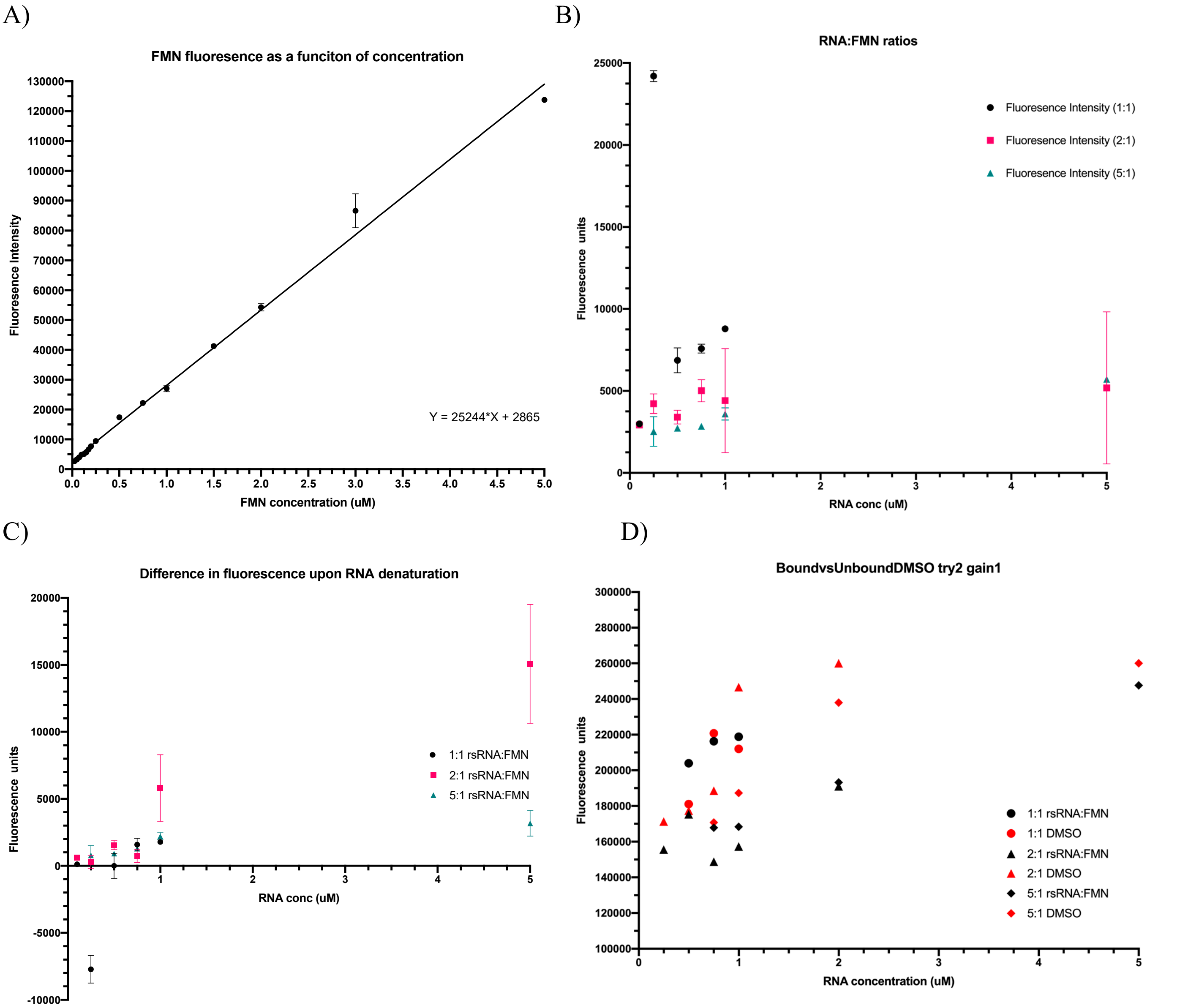

Figure S1. Fluorescence of FMN, FMN inbound to FRS, and of FMN and denatured FRS. A, B, and C, the symbols represent the average across 2 replicates, and the standard deviation is shown as error bars. D symbols represent a single replicate.

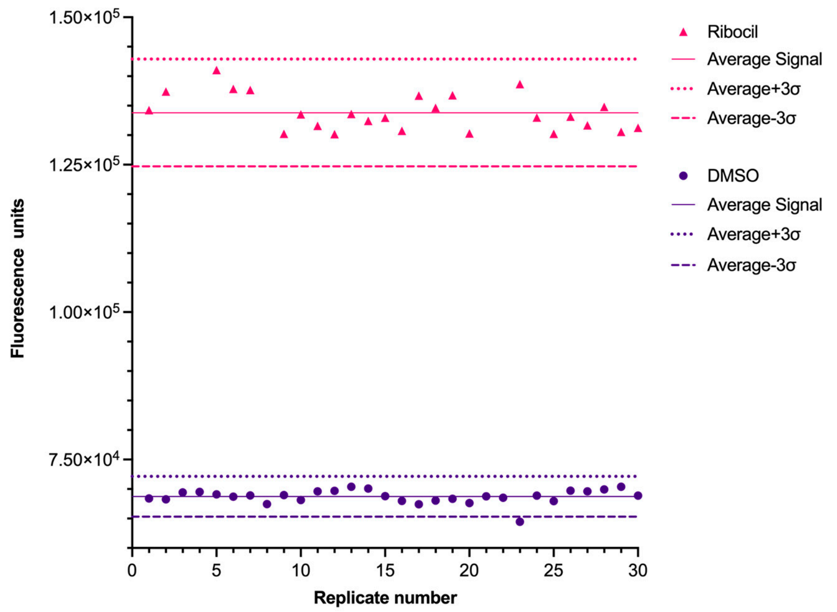

Figure S2. Robust investigation of fluorescence change upon equilibration with ribocil. Each triangle or circle represents a single replicate. Ribocil indicates 50 nL of DMSO and a final ribocil concentration of 10 uM in the 10.05uL of FMN and FRS solution. DMSO indicates 50 nL of DMSO in the 10.05uL of FMN and FRS solution. This assay was completed using an incubation time of 30 minutes and yielded an excellent Z' value of 0.82. While the difference in signal is small, the low standard deviations allow for the identification of molecules capable of interacting with the FRS.

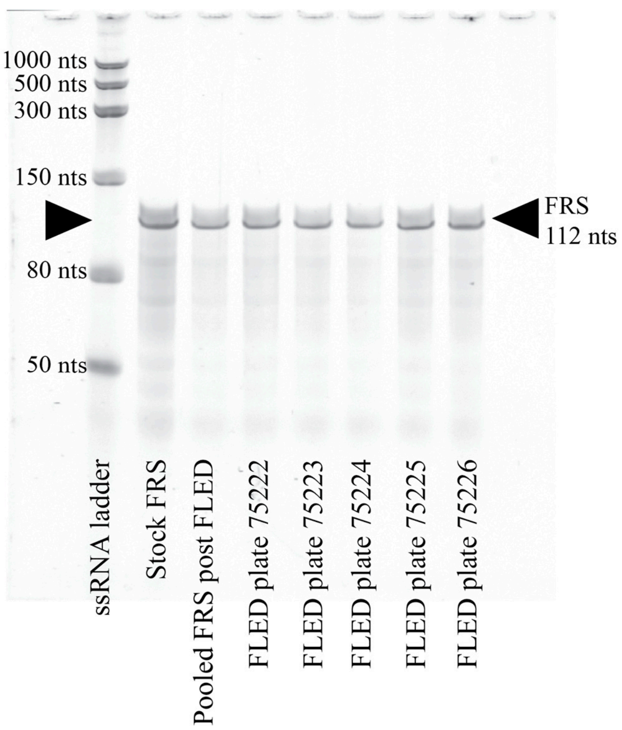

Figure S3. Representative RNA degradation post FLED testing. A 12% denaturing polyacrylamide gel of RNA samples after a Phase 1 FLED screening day. Each sample was a pooled mix of wells from the 384 plate and was diluted 30x. The control FRS sample was not subjected to refolding or held at room temperature but was diluted to a similar concentration.

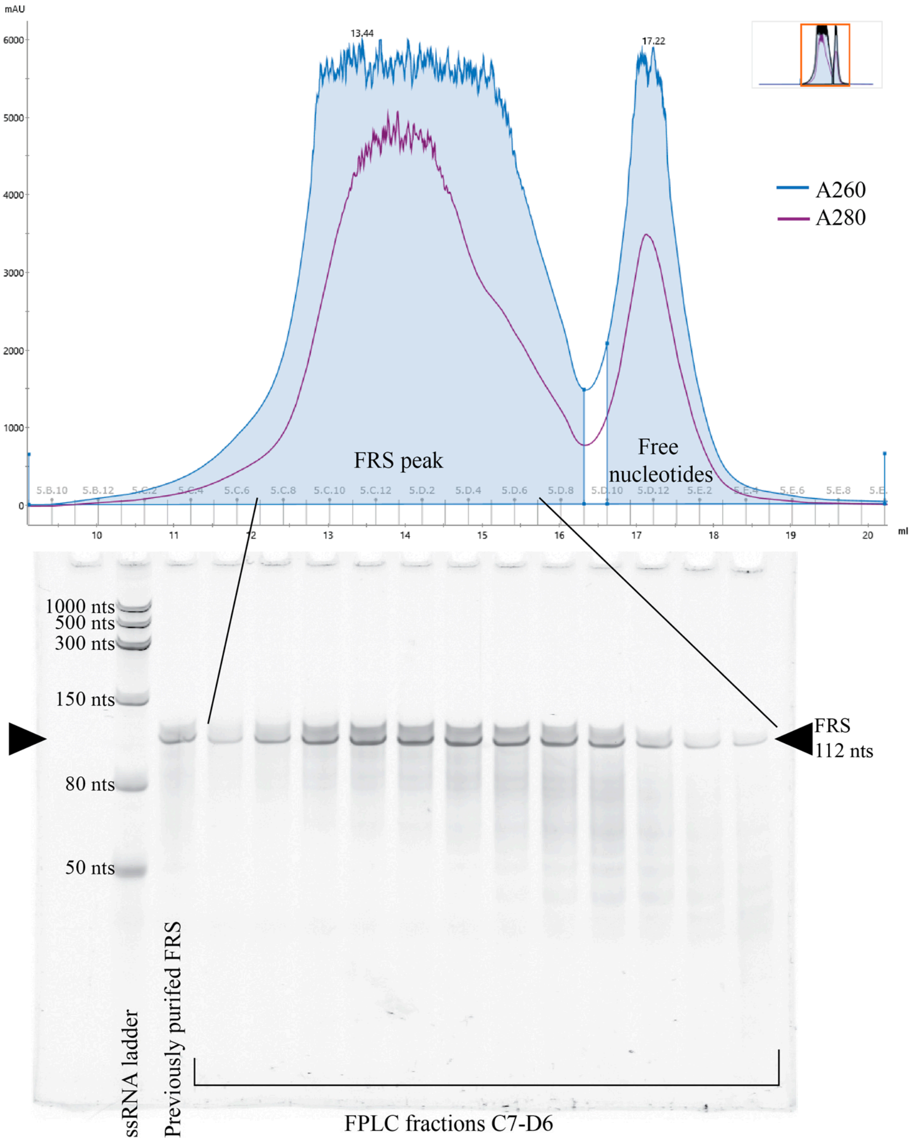

Figure S4. Representative FPLC purification of FRS. FPLC trace from AKTA Pure system aligned with 12% denaturing polyacrylamide gel.

Table S2. The concentration range for the 8-point dose-response testing of Phase 3 FLED hit compounds. Theoretical concentration is the programed concentration while the actual accounts for the limitations of the Echo liquid dispersion system.

Plate row	Theoretical concentration	Final concentration
1	100. µM	100. µM
2	50.0 µM	49.9 µM
3	25.0 µM	25.2 µM
4	12.5 µM	12.4 µM
5	6.25 µM	6.17 µM
6	3.13 µM	3.33 µM
7	1.56 µM	1.43 µM
8	0.781 µM	0.950 µM

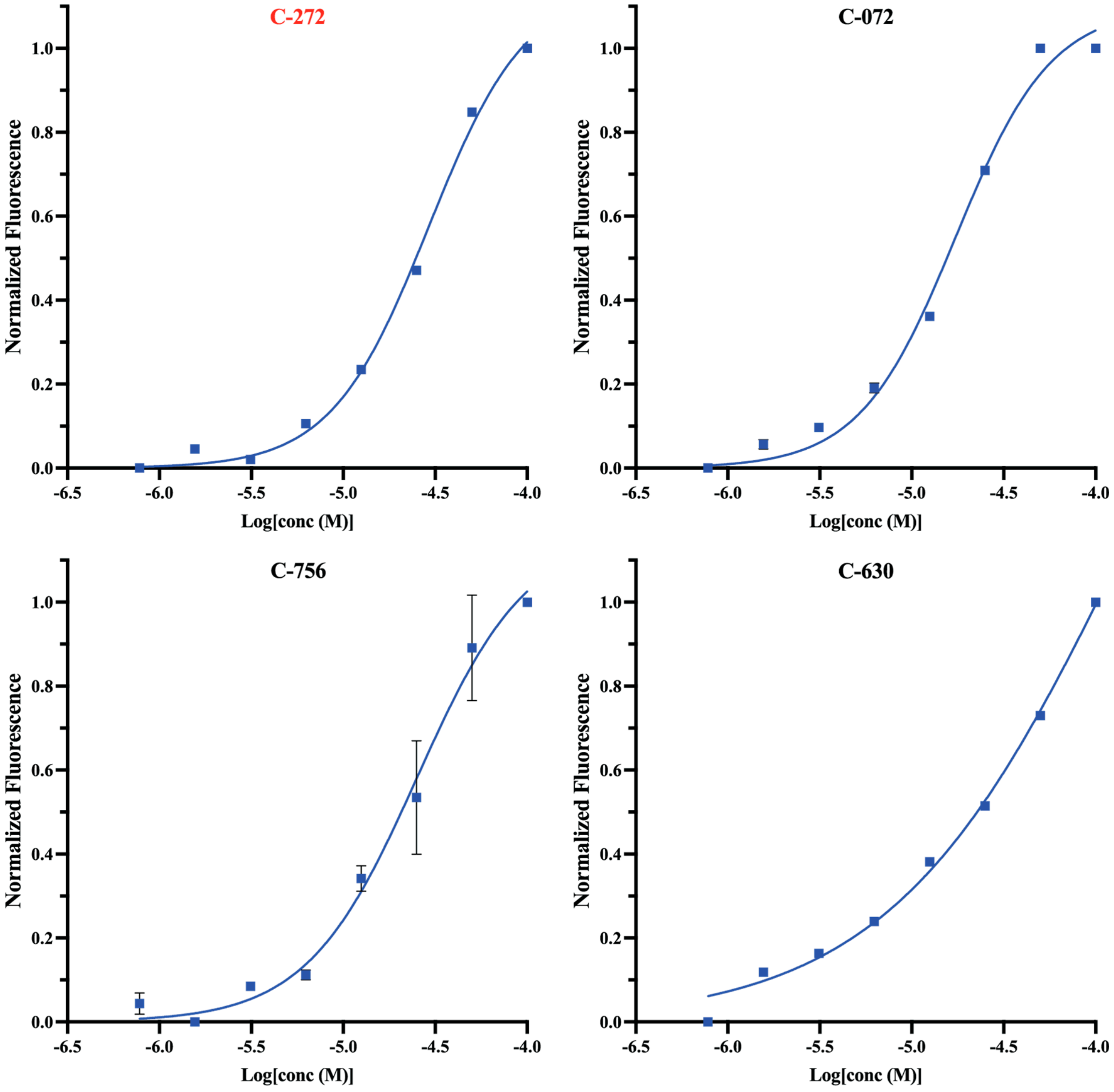

Figure S5. Further examples of concentration-response of FRS:FMN complex to SM. Four more hit compounds showed concentration-response effects. These compounds were previously included in the main text, but the compounds that are present on the representative plots for the first three phases of FLED replaced these plots in the main text. The compound title in red text was tested in Phase 3 and was still included in Phase 4.

Table S3. Example data from each phase of FLED screening. A compound ID was assigned to each molecule, and each phase is represented as a separate section. Both normalized and raw values are provided.

	Phase 1	
Compound ID	Raw fluorescence	Normalized fluorescence
C-587	103896	1.066
C-785	66549	0.432
	Phase 2	
Compound ID	Average raw fluorescence	Average normalized fluorescence
C-587	201890	1.788
C-785	147176	1.03
Phase 3		
Compound ID	Average raw fluorescence	Average normalized fluorescence
C-037	3750	0.047
C-410	37957	17.7
	Phase 4	

ID & concentration	Average raw fluorescence	Average normalized fluorescence
C-037 100 uM	260000	1
C-037 50.0 µM	260000	1
C-037 25.0 µM	171559	0.54
C-037 12.5 µM	110621	0.22
C-037 6.25 µM	100533	0.16
C-037 3.13 µM	72668	0.016
C-037 1.56 µM	79568	0.051
C-037 0.781 µM	72140	0.013
C-785 100 uM	192231	1
C-785 50.0 µM	172185	0.98
C-785 25.0 µM	176399	0.83
C-785 12.5 µM	147219	0.55
C-785 6.25 µM	119723	0.28
C-785 3.13 µM	104837	0.14
C-785 1.56 µM	94002	0.033
C-785 0.781 µM	90676	0.0
